# Gestational Perfluorooctanoic Acid Exposure Inhibits Placental Development by Dysregulation of Labyrinth Vessels and uNK Cells and Apoptosis in Mice

**DOI:** 10.3389/fphys.2020.00051

**Published:** 2020-02-07

**Authors:** Wenyu Jiang, Yu Deng, Zifan Song, Yajuan Xie, Lixin Gong, Yilu Chen, Haibin Kuang

**Affiliations:** ^1^Department of Physiology, Basic Medical College, Nanchang University, Nanchang, China; ^2^Department of Clinic Medicine, School of Queen Mary, Nanchang University, Nanchang, China; ^3^Jiangxi Provincial Key Laboratory of Reproductive Physiology and Pathology, Medical Experimental Teaching Center, Nanchang University, Nanchang, China

**Keywords:** perfluorooctanoic acid, placental development, toxicity, apoptosis, uNK cells

## Abstract

Perfluorooctanoic acid (PFOA) is a widely used perfluorinated compound and known to cause developmental toxicity which includes the increase of resorbed embryo, decrease of fetal survival, and fetal growth retardation. Nevertheless, whether it is associated with alteration of placental development remains unknown. Pregnant mice were gavaged with 0, 2.5, 5, 10 mg PFOA /kg/day from pregnancy day (PD) 1 to PD 13. Results showed that PFOA exposure markedly decreased the placental weight and caused interstitial edema of placenta. Laminin staining indicated that blood sinusoids area was shrunken in placenta of PFOA-exposed mice. Furthermore, PFOA treatment significantly reduced numbers of uNK cells. Western blot analysis revealed that levels of Bax and cleaved-caspase 3 proteins were markedly up-regulated in PFOA-treated groups. In addition, TEM examination showed that PFOA treatment caused rupture of nuclear membrane and nuclear pyknosis and fragmentation. Thus, our results suggested that gestational PFOA exposure significantly inhibited development of early placenta through shrinkage of labyrinth vessels and downregulation of uNK cells and apoptosis induction, which may result in adverse gestational outcomes.

## Introduction

Perfluorooctanoic acid (PFOA), a broadly used perfluorinated chemical, is extensively applied in industrial and consumer fields for super hydrophobic, oleophobic, and hydrophilic characteristics, such as fire-fighting foams, oil-resistant coatings, performance chemical, plumbing thread seal tape, emulsifier, and polishes (Kudo and Kawashima, 2003; Wang et al., 2015b). Due to the strongest carbon-fluorine bonds, PFOA was found to be able to resist environmental degradation like metabolism, hydrolysis, and photolysis (Liu et al., 2013; Wang et al., 2015b) and is extremely persistent in the natural world, including surface water, groundwater, house dust, food, and food packaging. Furthermore, it has also been detected in different parts of human body (Post et al., 2012). In 2009 Stockholm Convention, PFOA was listed as emerging persistent organic pollutant (Patterson et al., 2009).

Recently, PFOA has attracted more attentions for reproductive and developmental toxicities (Yahia et al., 2010; Das et al., 2015; Song et al., 2018). Experiments *in vivo* and *in vitro* showed that PFOA exposure reduced testosterone production through the down-regulation of steroid hormone related synthetase (Zhao et al., 2010a). In adult male mice, PFOA treatment for 14 consecutive days prominently damaged seminiferous tubules and decreased sperm numbers of testis and epididymis (Liu et al., 2015). Epidemiological analyses indicated that sperm aneuploidy and fragmented DNA markedly rose in PFOA-positive mans compared with negative group (Governini et al., 2015). In porcine ovarian granulosa and theca cells, PFOA administration dramatically decreased the secretion of basal and gonadotropin-stimulated steroid hormones (including progesterone, estradiol, and androstenedione) (Chaparro-Ortega et al., 2018). In the prospective birth cohort, serum PFOA levels in pregnant women were positively related to inhibin B concentration in the cord blood (Itoh et al., 2016). However, in human adrenocortical carcinoma cells, PFOA treatment had no significant effect on the testosterone and estradiol productions (Wang et al., 2015a). In addition, regression analyses indicated that menarche in the daughters with higher PFOA exposure were postponed 5.3 months compared with those with lower level exposure (Kristensen et al., 2013). Moreover, Lee’s study showed that PFOA levels in maternal blood were negatively correlated with newborn weight (Lee et al., 2013). During pregnancy, gestational exposure to PFOA obviously raised the numbers of resorbed embryo and reduced the number of survival offspring and fetal weight and caused fetal growth retardation in mice (Lau et al., 2006; Yahia et al., 2010; Suh et al., 2011; Chen et al., 2017; Caserta et al., 2018). Nevertheless, whether it is relevant to alteration of placental development remains unclear. Consequently, our aim of this study was to observe the effect and mechanism of maternal PFOA exposure on the growth and development of early placenta.

## Materials and Methods

### Chemicals and Reagents

PFOA (96% purity), biotinylated-dolichos biflorus (DBA) lectin, acetyl-D-galactosamine, and laminin antibody were obtained from MilliporeSigma Chemical Company (St. Louis, MO, USA). Rabbit anti-β-actin, Bax, and cleaved-caspase 3 were purchased from Cell Signaling Technology (MA, USA). Streptavidin-peroxidase and diaminobenzidine solution were provided by Zhongshan Biotechnology Co., Ltd. (Beijing, China). Phosphatase inhibitor cocktail and polyvinylidene difluoride (PVDF) membrane were bought from Applygen Technologies (Beijing, China). All other chemicals were obtained from Nanchang preeminent biology Co., Ltd. (Nanchang, China).

### Animals and Treatment

Adult Kunming mice (25–30 g) were purchased from the Laboratory Animal Center of Jiangxi traditional Medical University. Mice were housed at room temperature with a 12 h light/dark cycle with free access to food and water. Female mice were bred with fertile male at the ratio of 2:1. In the next morning, all females were checked for vaginal plug and the presence was defined as pregnancy day (PD) 1. Dams were separated into four groups (*n* = 6/group) and exposed with PFOA (0, 2.5, 5, 10 mg/kg/day) daily by oral gavage. Control group was treated with deionized water. Experimental animals were anesthetized with pentobarbital sodium prior to cervical dislocation and uterine collection at about 16:00 on PD 13. Embryos and placentas from these mice were weighed and taken photos by digital camera (Nikon, Japan) and were frozen in liquid nitrogen for further research. This study was performed in accordance with guidelines approved by the Animal Ethics Committee of Nanchang University. All mice were treated humanely according to the guidelines for laboratory animal science at Nanchang University.

### Hematoxylin and Eosin Staining

Placentas were fixed in Bouin’s solution, kept in gradient ethanol solution (70, 80, 95% twice and 100% twice), and cleared with xylol. Sections were stored at 4°C for the histomorphology and immunohistochemistry analysis. Sections were stained with Hematoxylin and Eosin (H&E) for morphological observation. The areas of spongiotrophoblast and labyrinth and whole placenta were counted using sections with the maximum parts for the layer of whole placenta by Image J software (NIH, USA). Mean area for per group was calculated using five serial sections from six individuals.

### Immunohistochemistry

Placental tissues were deparaffinized and rehydrated in xylol and descending ethanol solutions, respectively. Non-specific binding was treated with 5% BSA in PBS for 30 min, and then the samples were mixed with rabbit anti-laminin (1:200) or biotinylated DBA-lectin (1:1200) for overnight at 4°C. After washing with PBS, the sections were incubated with secondary antibody for 60 min at 37°C. Positive signals were indicated with diaminobenzidine solution. The numbers of cells positive for DBA lectin staining were counted in 10 non-overlapping fields at magnification, ×400.

### Western Blot Analysis

Placental tissues were homogenized in lysis buffer containing the phosphatase inhibitor cocktail and PMSF and then centrifuged 15,000*g* for 15 min at 4°C for protein extraction. Proteins (20 μg per sample) in loading buffer were loaded to 15% SDS-PAGE gel for electrophoresis and blotted onto a PVDF membrane. The membrane was mixed with 5% skim milk for 1 h at room temperature and incubated with the primary antibodies overnight at 4°C. After washing in TBST solution, the blots were then incubated with goat anti-rabbit secondary antibodies (1:5,000) for 1 h at room temperature. Immunoreactive signal was observed by enhanced chemiluminescence (ECL) detection kit. The expression levels of proteins were determined by the densitometry of protein bands using Quantity One software and normalized to β-actin expression.

### Transmission Electron Microscopy Analyses

Placenta tissues (about 1 mm^3^ in size) were fixed in ice-cold 2.5% glutaraldehyde phosphate buffer overnight and 1% osmium acid for 1 h at room temperature and were dehydrated in an ascending ethanol (50, 70, 90, and 100%) solution and 100% acetone. Then tissues were embedded in Epon 812, solidified and sectioned at 120 nm, and were double stained with 4% uranyl acetate and lead citrate. Representative parts were observed with a TECNAI 10 TEM (Philips, Nederland). Five fields were randomly selected from each sample for apoptosis analysis (based on the changes in nuclear morphology).

### Statistical Analysis

All statistical analyses were carried out using GraphPad Prime 5 software (La Jolla, CA). The data are presented as the means ± standard error (SE) and checked by Shapiro-Wilk test, and compared with one-way analysis of variance (ANOVA) followed by LSD’s *post-hoc* test or using Student’s *t*-test between two groups. Levels of significant difference were set at *p* < 0.05.

## Results

### Maternal Perfluorooctanoic Acid Exposure Reduced the Weight of Early Placenta

**Figure 1:**
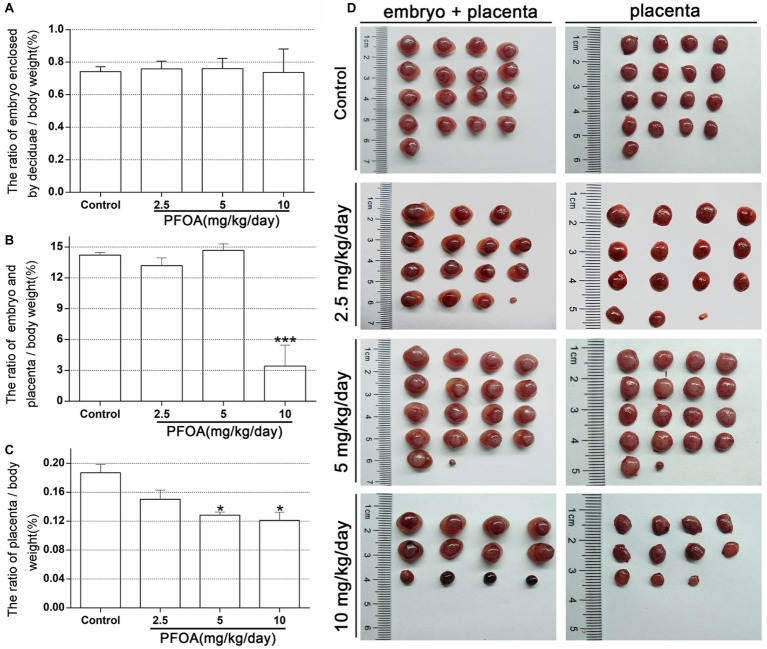
Maternal PFOA exposure reduced the weight of early placenta. **(A)** The ratio of embryo enclosed by decidua to the body weight. **(B)** The ratio of embryo plus placenta to the body weight. **(C)** The ratio of placenta to the body weight. **(D)** Representative pictures of embryo and placenta (a single litter shown for each group) collected on gestational days 13. Values are represented as the mean ± SE (*n* = 6 mice/treatment group). ^*^p < 0.05, ^***^p < 0.001 compared with control.

There is no significant difference in the ratio of embryo enclosed by deciduas to the body weight between PFOA-treated groups and control group (Figure 1A). However, the ratio of embryo and placenta to the body weight decreased by 74.23% after treatment with 10 mg/kg/day PFOA (Figures 1B,D). Furthermore, 5 and 10 mg/kg/day PFOA treatment also markedly reduced the ratio of placenta to body weight (Figures 1C,D).

### Maternal Perfluorooctanoic Acid Exposure Disturbed Placental Histology

**Figure 2:**
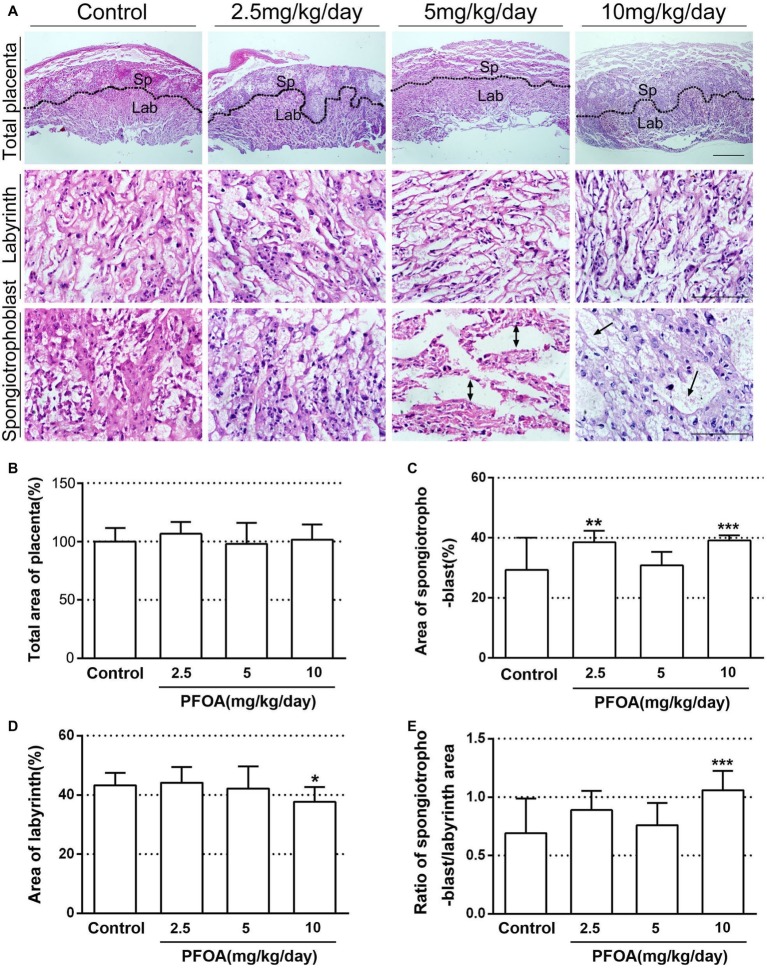
Histopathological changes of placenta after PFOA exposure. **(A)** Hematoxylin and eosin staining of placenta sections. **(B)** Total area of placenta (%). **(C)** The ratio of spongiotrophoblast area to total area (%). **(D)** The ratio of labyrinth area to total area (%). **(E)** The ratio of spongiotrophoblast area to labyrinth area. Scale bar, 50 μm. Values are represented as the mean ± SE, *n* = 6. ^*^p < 0.05, ^**^p < 0.01, ^***^p < 0.001 compared with control.

Placental histology indicated that PFOA exposure induced obvious alterations in the placentas of pregnant mice. As shown in Figure 2A, spongiotrophoblast in 5 and 10 mg/kg/day PFOA groups exhibited interstitial edema of placenta. No significant variation in the total area of placenta was found in all experimental mice (Figure 2B). However, low and high doses of PFOA cause a prominent increase in the areas of spongiotrophoblast to the total area (Figure 2C). PFOA exposure showed no significant effect on the ratio of labyrinth area to the total area (Figure 2D), but high-dose (10 mg/kg/day) PFOA treatment dramatically decreased labyrinth area, and increased the ratio spongiotrophoblast area to labyrinth area compared to control group (Figure 2E).

### Perfluorooctanoic Acid Exposure Resulted in the Shutting of Vascular Lumen and Reduced the Numbers of uNK Cells

**Figure 3:**
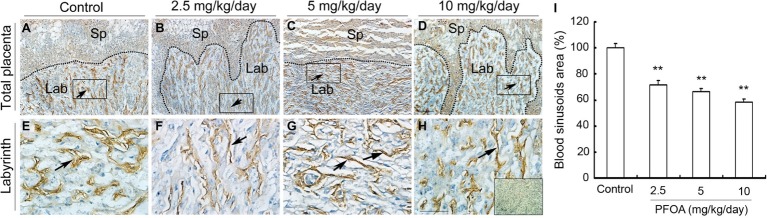
The effect of PFOA exposure on the labyrinth vessel of placenta. The representative immunohistochemical staining of laminin showed blood vessel changes of placental labyrinth collected from mice which were administrated by control **(A)**, 2.5 **(B)**, 5 **(C)**, and 10 **(D)** mg/kg/day PFOA. Rectangular areas in **(A–D)** (original magnification ×100) were magnified in the bottom **(E,F)** with higher magnification of ×400. The inset of **(H)** is negative control. Arrows: blood vessels. Sp: spongiotrophoblast; Lab: labyrinth. Scale bar, 50 μm. **(I)** Blood sinusoids area in the labyrinth region. Values are represented as the mean ± SE (*n* = 12 placentas/6 mice/treatment group). ^**^p < 0.01 compared with control.

**Figure 4:**
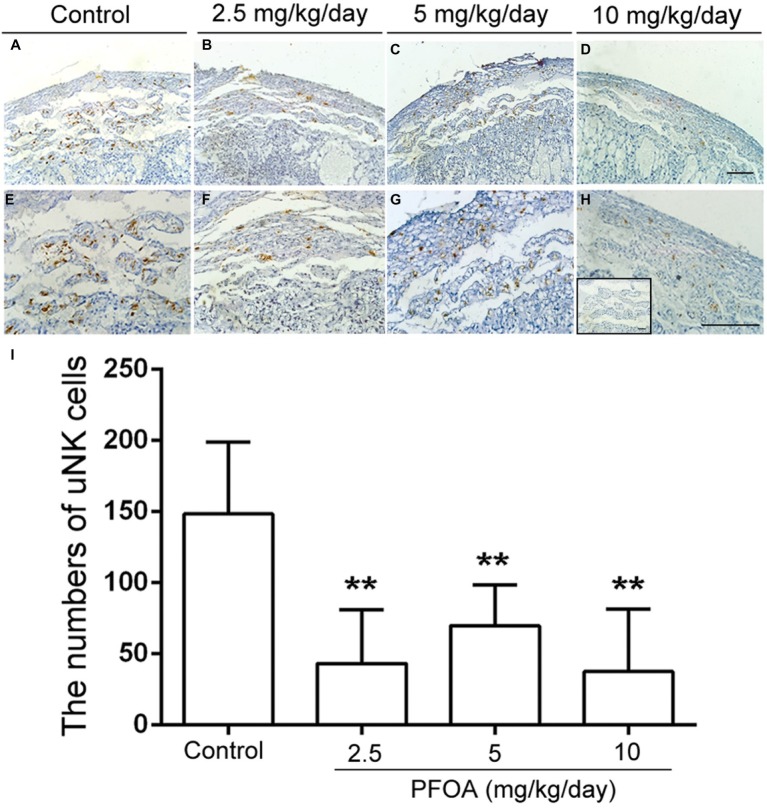
PFOA exposure down-regulated the numbers of uNK cells. DAB staining indicated the changes of uNK cells in the deciduas collected from mice which were administrated by control **(A)**, 2.5 **(B)**, 5 **(C)**, and 10 **(D)** mg/kg/day PFOA. Images in the upper panels (**A–D**, original magnification×100) were magnified in the bottom **(E–H)** with higher magnification of ×200, respectively. The inset of **(H)** is negative control. Scale bar, 50 μm. **(I)** The numbers of uNK cells. Values are represented as the mean ± SE (*n* = 6 placentas/6 mice/treatment group). ^**^p < 0.01 compared with control.

Laminin staining showed there are lots of fetal vessels in the placental labyrinth of control group, which are regularly distributed and form intricate and organized branching network and its lumen are dilated (Figures 3A,E). However, administration of different doses PFOA (2.5–10 mg/kg/day) significantly reduced blood sinusoids area and shutting of vascular lumen (Figures 3B–D,F). Furthermore, all doses PFOA exposure significantly reduced the amounts of uNK cells in the deciduas of placenta (Figure 4).

### Maternal Perfluorooctanoic Acid Exposure Induced Placental Apoptosis

**Figure 5:**
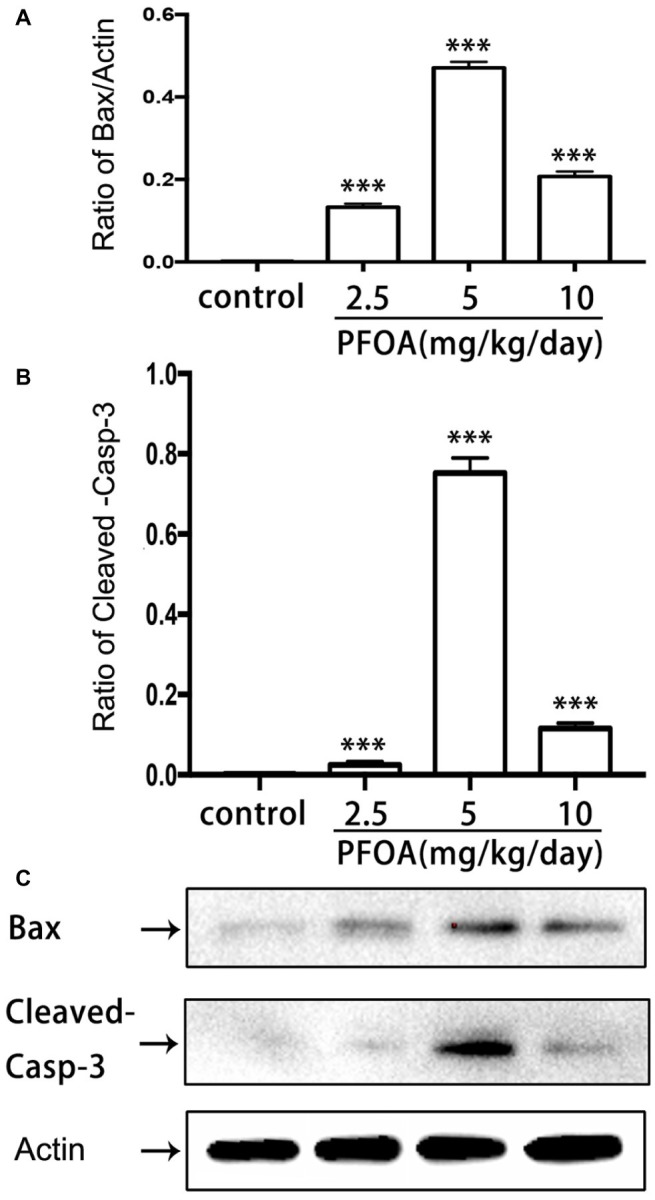
Effect of PFOA exposure on apoptosis-related proteins of placenta. **(A)** Relative expression levels of Bax protein. **(B)** Relative expression levels of cleaved-caspase 3 protein. **(C)** Representative western-blotting images of Bax and cleaved-caspase 3 proteins. Data are normalized to β-actin and represented as the mean ± SE, *n* = 6. ^***^p < 0.001 compared with control.

**Figure 6:**
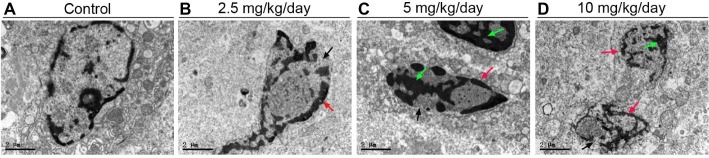
TEM analyses of placental tissues after PFOA exposure. The representative TEM micrographs of the placental tissues collected from mice which were administrated by control **(A)**, 2.5 **(B)**, 5 **(C)**, and 10 **(D)** mg/kg/day PFOA. Black arrows indicate nuclei. Scale bar, 2 μm.

Blotted results revealed that expression levels of Bax (Figures 5A,C) and cleaved-caspase 3 proteins (Figures 5B,C) were markedly up-regulated in all three doses PFOA-treated groups compare control group, with a maximal increase observed at 5 mg/kg/day PFOA. Moreover, TUNEL staining indicated that 2.5, 5, 10 mg/kg/day PFOA significantly increased apoptotic positive cell numbers of placental tissues (Supplementary Figure 1). TEM examination showed that PFOA treatment caused extensive morphological changes of nucleus in the placental cells, which were characterized by the rupture of nuclear membrane, nuclear pyknosis and fragmentation, and chromosome condensation of placental cells (Figure 6).

## Discussion

Previous studies indicated that PFOA exposure obviously increased the numbers of resorbed embryo and suppressed fetal growth during pregnancy in human and mice (Koustas et al., 2014; Chen et al., 2017). Our results showed for the first time gestational PFOA exposure markedly inhibited the development of early placenta *via* shrinkage of labyrinth vessels and downregulation of uNK cells and apoptosis induction in mice, which has possibly contributed to adverse pregnancy outcomes such as early pregnancy loss and decrease of fetal growth. 5 and 10 mg/kg/day PFOA treatment dramatically decreased placental relative weight and resulted in the interstitial edema of placenta. Immunohistochemical staining evidenced that PFOA exposure caused the shutting of fetal vessels and down-regulation of uNK cell numbers in the deciduas of placenta. Western blot results revealed that PFOA exposure significantly increased apoptosis-related protein Bax and cleaved-caspase 3 levels. Furthermore, TEM examination found that PFOA treatment induced the rupture of nuclear membrane and nuclear pyknosis and fragmentation of placental cells.

The placenta is a special and important organ during gestation, which can provide enough oxygen and nutrients for the growth and development of fetus (Papanikolaou et al., 2018). In the present study, maternal exposure to PFOA markedly decreased placental weight and induced the interstitial edema of placental spongiotrophoblast, which probably resulted in fetal resorptions and growth retardation and compromised postnatal survival. Song et al.’s studies showed that placental weight and the numbers of survival offspring mice were dramatically reduced in the PFOA-treated groups (Lau et al., 2006; Yahia et al., 2010; Song et al., 2018). This result was almost in accord with our current and previous research (Chen et al., 2017). Furthermore, Caserta D reported that PFOA levels in maternal blood were negatively related to newborn weight (Caserta et al., 2018). Prenatal exposure to perfluorooctanesulfonicacid (PFOS) and PFOA can be transferred from mother to fetus through the placental barrier and is considered to affect the development of human fetus (Mamsen et al., 2017).

Studies demonstrated that aberrant placental angiogenesis was associated with fetal growth and neonatal body weight and survival rate (Torry et al., 2004). Results from this study showed that microvessel space in the labyrinthine region was shrunken and shut in placenta of PFOA-treated mice, suggesting disorder of placenta exposure to PFOA may be caused by dysfunction of the vascular structure. It was found that PFOS treatment resulted in the dilatation of fetal intracranial blood vessel along with severe lung collapse which led to neonatal mortality during mice pregnancy (Yahia et al., 2008). Furthermore, Liu’s study evidenced that PFOA significantly increased paracellular permeability of human retina endothelial cells through the degradation of adherens junctions (Liu et al., 2018). However, Spachmo B did not find that PFOS and PFOA exposure significantly damaged the angiogenesis of Atlantic salmon embryos and larvae (Spachmo and Arukwe, 2012). In addition, PFOA-exposure mice did not show intracranial blood vessel dilatation although 5 and 10 mg/kg PFOA obviously attenuated the neonatal survival rate (Yahia et al., 2010). Therefore, the cause and mechanism of neonatal death by PFOA may be diverse from PFOS and are the areas of future study.

uNK cells are the most plentiful granulated lymphocyte population in the maternal-feto interface during pregnancy (Gong et al., 2017). They secrete abundant cytokines and regulate trophoblast invasion, vascular remodeling, and placental development which are vital to success pregnancy (Tripathi et al., 2015; Gong et al., 2017). Our results showed that PFOA exposure significantly reduced the numbers of uNK cells, which is a plausible explanation for pregnancy loss. Previous study indicated that estradiol could alter the homing, development, and physiological function of uNK cells (Gong et al., 2017). PFOA is an estrogen-like effect of environmental endocrine-disrupting chemicals (Zhao et al., 2010b), and our previous study demonstrated that PFOA exposure significantly suppressed luteal function by oxidative stress and apoptotic pathway in mice during pregnancy (Chen et al., 2017). Therefore, down-regulation of uNK cell numbers in the deciduas of placenta may be caused by changes of estrogen and progesterone in PFOA-treated mice.

The apoptosis plays an important role in the growth and development of placenta. Bax, a pro-apoptotic member of the Bcl-2 protein group, can facilitate the release of cytochrome c from mitochondria and then trigger apoptosis progress (Jurgensmeier et al., 1998). PFOA administration in our study sharply increased the expression of Bax protein. It is consistent with Liu’s study, in which levels of Bax and p-p53 proteins obviously increased, and Bcl-2 protein significantly decreased in the testis of PFOA-treated mice (Liu et al., 2015). In zebrafish liver cells, the level of Bax mRNA also significantly increased in the PFOS exposure, but not PFOA exposure (Cui et al., 2015). Besides, members of the caspase family of aspartic acid-directed cysteine proteases lead to cell apoptosis by means of flawing the cellular structure and function (Ratts et al., 2000; Cui et al., 2015). Among caspase family, caspase-3 is a central effector caspase in many cells and mediates the cleavage of itself, other caspase and downstream substrates (Gown and Willingham, 2002). Our results evidenced PFOA treatment significantly increased expression of cleaved caspase-3 protein. In addition, both PFOS and PFOA treatment can significantly induce the activation of caspase-3, -8, and -9 in primary cultured hepatocytes. However, specific signal pathway of PFOA-induced placental apoptosis requires further investigation in mice.

In summary, our study observed the effects of maternal PFOA exposure on the placental growth and development and evidenced for the first time that gestational PFOA exposure markedly inhibited the growth and development of early placenta through labyrinth vessels shrinkage and decreased uNK cells and apoptosis induction, which probably resulted in adverse gestational outcomes. Our results will be beneficial to promoting continuous attention about the health risk from high exposure to PFOA for the pregnant women.

## Data Availability Statement

All datasets generated for this study are included in the article/Supplementary Material.

## Ethics Statement

The animal study was reviewed and approved by the Animal Ethics Committee of Nanchang University.

## Author Contributions

WJ, YD, and ZS contributed equally to this study. HK and YC were responsible for conceptualization. WJ, YD, ZS, YX, and LG were responsible for experimental investigation. ZS was responsible for data curation. YD, YX, and LG were mainly responsible for data processing and picture modification. WJ, YD, YX, and LG were responsible for writing-original draft preparation. HK proofread the final manuscript before submission. All authors read and approved the final manuscript.

### Conflict of Interest

The authors declare that the research was conducted in the absence of any commercial or financial relationships that could be construed as a potential conflict of interest.
