# Supplementary figures and images for: Gestational Perfluorooctanoic Acid Exposure Inhibits Placental Development by Dysregulation of Labyrinth Vessels and uNK Cells and Apoptosis in Mice
Source: Front Physiol. 2020 Feb 7;11:51. doi: 10.3389/fphys.2020.00051 (PMC7025578; doi:10.3389/fphys.2020.00051)

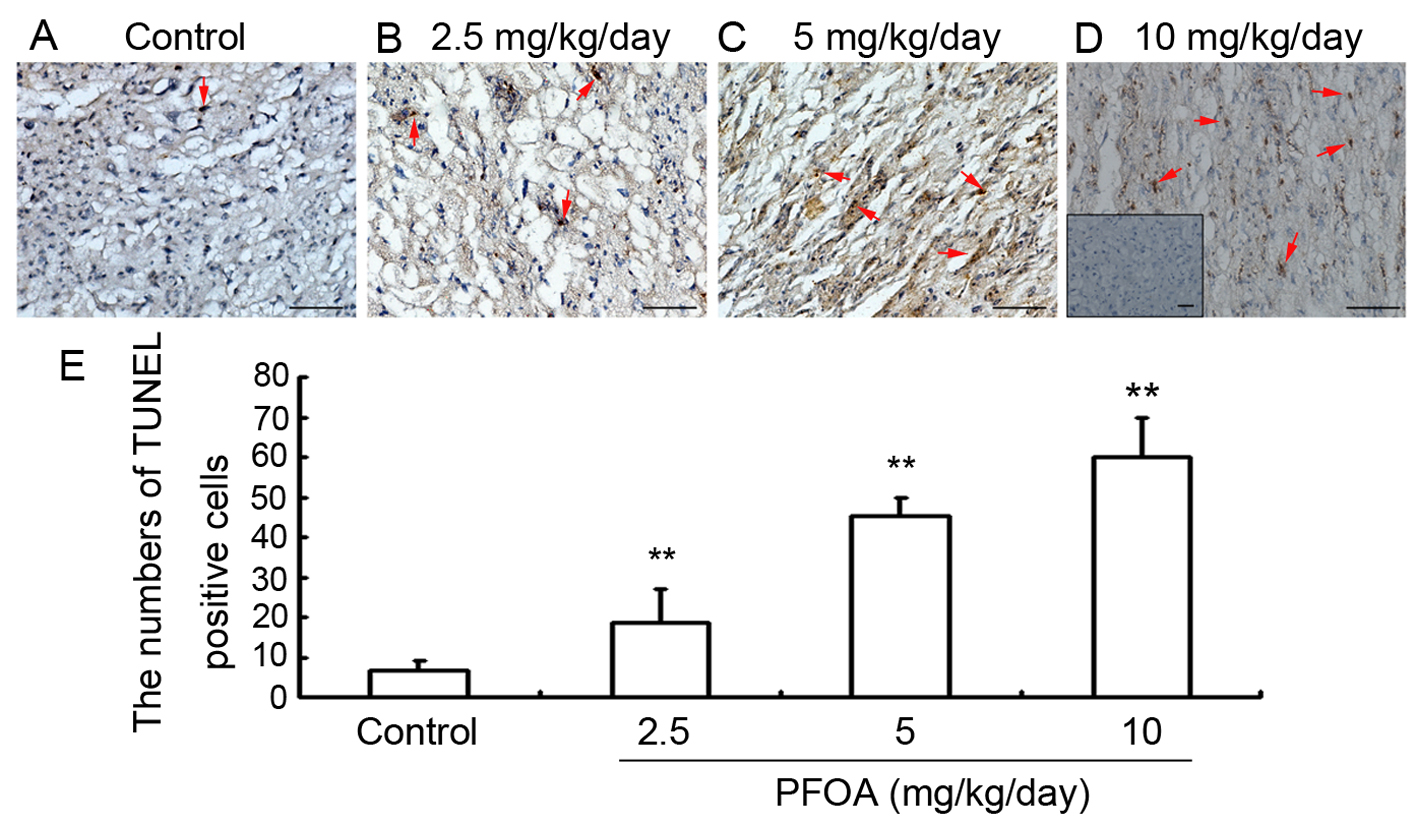

Supplement: Supplementary file 1 [file Image_1.JPEG]
